# Tolerability of Sodium-Glucose Co-transporter 2 Inhibitors in Patients With Type 2 Diabetes Mellitus: A Retrospective Cohort Study

**DOI:** 10.7759/cureus.75798

**Published:** 2024-12-16

**Authors:** Suhaib Radi, Maher Almutairi, Abdullah S Alghamdi, Mustafa Alzahrani, Siraj Alghamdi, Nawwaf Almalky, Bassam Alharbi, Talal M Altuwaylie, Fawziah Marwani, Wafa Saber

**Affiliations:** 1 College of Medicine, King Saud Bin Abdulaziz University for Health Sciences, Jeddah, SAU; 2 Department of Medicine, King Abdullah International Medical Research Center, Jeddah, SAU; 3 Division of Endocrinology, Department of Internal Medicine, Ministry of the National Guard-Health Affairs, Jeddah, SAU; 4 Department of Research, King Abdullah International Medical Research Center, Jeddah, SAU; 5 Department of Medicine, King Faisal Specialist Hospital and Research Center, Jeddah, SAU

**Keywords:** diabetes mellitus, genital infections, sodium-glucose co-transporter 2 inhibitors, type 2 diabetes, urinary tract infection

## Abstract

Background

Sodium-glucose co-transporter 2 (SGLT2) inhibitors are an emerging treatment for type 2 diabetes mellitus (T2DM). The effect and tolerability of SGLT2 inhibitors in patients with T2DM, especially related risk factors and susceptible populations, are an area of ongoing research.

Aim

The aim of this study was to evaluate the tolerability of SGLT2 inhibitors, particularly the risk associated with urogenital infection, in patients with T2DM.

Methods

This retrospective cohort study included 275 participants (median age: 64 years). Electronic medical records of patients with T2DM who underwent treatment with approved SGLT2 inhibitors between January 2020 and December 2022 at our institute were reviewed. Multiple variables were used to assess the tolerability of SGLT2 inhibitors and factors associated with genitourinary infections.

Results

The incidence of genitourinary infections was 13.1% among patients with T2DM taking SGLT2 inhibitors, which was lower than the reported national and global incidences of urinary tract infections (UTIs) among patients with diabetes. Patients with a history of UTIs were more likely to develop recurrent infections (hazard ratio [HR], 3.32; 95% confidence interval [CI]: 1.56, 7.09). A higher pretreatment glomerular filtration rate was associated with a lower risk of UTIs (HR, 0.98; 95% CI: 0.97, 0.99).

Conclusions

SGLT2 inhibitor administration in patients with T2DM does not significantly increase the risk of UTIs compared with the reported national and global rates of UTIs among patients with diabetes. Variable hygiene practices among the Muslim community may be a possible explanation for the observed differences in the rates of UTIs.

## Introduction

Type 2 diabetes mellitus (T2DM) is a progressive endocrine and metabolic disorder that poses a substantial global health challenge, affects millions of individuals, and is anticipated to affect approximately 592 million people by 2035 [1]. Given the multifaceted nature of T2DM development, diverse therapeutic modalities have been devised. The emergence of sodium-glucose co-transporter 2 (SGLT2) inhibitors has significantly advanced the management of T2DM. SGLT2 inhibitors function by targeting the proximal renal tubules, inhibiting glucose reabsorption, and consequently elevating glucose excretion in the urine [2]. This mechanism not only aids the reduction of blood glucose levels but also holds the potential for ancillary benefits, including weight loss and blood pressure reduction. In addition, SGLT2 inhibitors enhance cardiovascular outcomes and mitigate secondary effects such as heart failure and renal disease progression in individuals diagnosed with pre-existing cardiovascular disease or chronic kidney disease [3].

Consequently, SGLT2 inhibitors are increasingly being used for the treatment of T2DM. However, a meta-analysis reported a higher incidence of urinary tract infections (UTIs) among patients receiving SGLT2 inhibitors compared with those administered a placebo (odds ratio [OR]: 1.34, 95% confidence interval [CI]: 1.03-1.74). Additionally, a significant elevation in the occurrence of genital infections was observed in patients using SGLT2 inhibitors compared with those using other hypoglycemic medications (OR: 5.06, 95% CI: 3.44-7.45) [4]. This phenomenon may be attributed to glucosuria, which creates an environment conducive to bacterial and fungal proliferation. Furthermore, a comprehensive understanding of the underlying mechanisms requires investigations of the interactions between multiple factors, including immunological responses, alterations in vaginal and urethral pH, and the presence of comorbidities [5].

The precise risk of urogenital infections associated with SGLT2 inhibitor use and the specific patient populations most susceptible to these complications remain unclear; moreover, no local studies have investigated this risk. Therefore, we conducted this first local study to evaluate the tolerability of SGLT2 inhibitors and risk factors contributing to the increased risk of urogenital infections in patients with T2DM.

## Materials and methods

Study design and participants

This retrospective cohort chart review-based study, conducted at the King Abdulaziz Medical City in Jeddah, Saudi Arabia, included individuals of both sexes, aged ≥18 years, diagnosed with T2DM, and undergoing treatment with SGLT2 inhibitors approved in Saudi Arabia (dapagliflozin, empagliflozin, and canagliflozin). The inclusion criterion was a minimum follow-up duration of one year after the initiation of SGLT2 inhibitor treatment between January 2020 and December 2022. Patients with a history of recurrent urogenital infections before SGLT2 inhibitor initiation were excluded.

All procedures followed were in accordance with the ethical standards of the ethics committee of the King Abdullah Medical Research Center, study number NRJ22J/009/01, dated March 10, 2022, and/or with the Helsinki Declaration of 1964 and later versions. Informed consent or substitute for it was obtained from all patients for being included in the study.

Sample size determination, sampling technique, and data collection

In the sample size calculation, using Raosoft's sample size calculator, an estimated annual T2DM caseload of 1,000 cases at the institute was considered. With a 5% margin of error, 95% CI, and 50% response distribution, the minimum sample size required for this study was 278 patients [6]. Convenience sampling was used for participant selection. The data collection tool developed by the principal investigator was validated, approved by the Institutional Review Board, and administered by the research team. Information was extracted from the electronic health record system in our center, BestCare 2.0, developed by Seoul National University Bundang Hospital. Data regarding the following variables were collected: medical record number, age, sex, the type and duration of T2DM, the specific SGLT2 inhibitor used, other antidiabetic agents used, the dosage and frequency of medication, urogenital symptoms, and other potential side effects, including euglycemic diabetic ketoacidosis, hypotension, acute kidney injury, amputation, and bone fractures.

Statistical analysis

The sociodemographic characteristics of the participants were assessed using descriptive and summary statistics. Continuous variables are expressed as medians (interquartile ranges), while categorical variables are presented as numbers and percentages. The Wilcoxon rank sum test was used to compare numerical and categorical variables, while Pearson's chi-squared test was used to compare categorical variables. A p-value of <0.05 was considered statistically significant. Risk factors for UTIs were assessed using hazard ratios (HRs) with 95% CIs, and the log-rank test was used to identify the likelihood of developing UTIs associated with SGLT2 inhibitor use. The Kaplan-Meier curve was used to compare the development of urogenital infections associated with SGLT-inhibitor between both sexes. The data were analyzed using JMP (version 10.0m SAS Institute Inc., Cary, NC, USA).

## Results

A total of 275 participants (median age: 64 years) were included in the study. Among them, 53% were women, 79% had hypertension, and 78% had dyslipidemia. Approximately half of the participants (52%) had evident atherosclerotic cardiovascular disease, and 20% had diabetic nephropathy. Additionally, 24% of the participants had a history of UTIs, and 6.9% had a history of genital infections (Table 1).

**Table 1:** Demographic characteristics of the study participants ^1^Data are presented as n (%) or median (interquartile range) BMI, body mass index; GFR, glomerular filtration rate

Characteristic	N = 275^1^
Sex	
Female	145 (53%)
Male	129 (47%)
Age (years)	64 (56, 72)
Chronic diseases	
Hypertension	218 (79%)
Dyslipidemia	215 (78%)
Atherosclerotic cardiovascular disease	143 (52%)
Nephropathy	56 (20%)
Retinopathy	45 (16%)
Cancer	17 (6.2%)
Baseline hemoglobin A1c (%)	8.80% (7.60%, 10.70%)
Baseline GFR (mL/min/1.73 m^2^)	80 mL/min/1.73 m^2^ (58, 95)
Baseline BMI (kg/m^2^)	32.8 kg/m^2^ (28.3, 36.8)
History of genital infections	
No	243 (93%)
Yes	18 (6.9%)
History of urinary tract infection	
No	203 (76%)
Yes	63 (24%)

The median baseline hemoglobin A1c levels were 8.8% before the initiation of SGLT2 inhibitors and 8.3% after the discontinuation of the inhibitor. Similarly, the median baseline glomerular filtration rate (GFR) was 80 mL/min/1.73 m^2^, which increased to 81 mL/min/1.73 m^2^ after the discontinuation of SGLT2 inhibitors. The baseline body mass index (BMI) was 32.8 kg/m^2^, which decreased to 31.1 kg/m_2_ after SGLT2 inhibitor discontinuation.

In total, 36 (13.1%) patients developed urogenital infections after the initiation of SGLT2 inhibitors. Among those with a prior history of UTIs, 28.4% experienced recurrent infections following SGLT2-inhibitor initiation. Among the patients who developed urogenital infections, 19.4% stopped using SGLT2.

Notably, there were significant differences in the history of genital infections and UTIs between men and women. Women had a higher frequency of a history of UTIs (p<0.001) and genital infections (p=0.001) (Table 2). Furthermore, women were more prone to developing UTIs after using SGLT2 inhibitors than men (p=0.004) (Table 3).

**Table 2:** Comparison of baseline characteristics between men and women ^1^Data are presented as n (%) or median (interquartile range) ^2^Wilcoxon rank sum test; Pearson's chi-squared test A p-value of <0.05 was considered statistically significant. DDP4, dipeptidyl peptidase 4; GLP1, glucagon-like peptide-1

Characteristic	Female, N = 145^1^	Male, N = 129^1^	p-Value^2^
Age	64 (59-72)	64 (55-72)	0.4
Duration of diabetes in years	20 (14-24)	20 (12-23)	0.9
History of prior genital infections			
No	120 (88%)	122 (98%)	0.001
Yes	16 (12%)	2 (1.6%)	
History of urinary tract infection			
No	90 (64%)	112 (90%)	<0.001
Yes	51 (36%)	12 (9.7%)	
The use of antidiabetic agents			
Insulin			
No	30 (21%)	30 (23%)	0.6
Yes	115 (79%)	99 (77%)	
Metformin			
No	46 (32%)	52 (40%)	0.14
Yes	99 (68%)	77 (60%)	
DDP4 inhibitors			
No	101 (70%)	103 (80%)	0.054
Yes	44 (30%)	26 (20%)	
GLP1 agonist			
No	91 (63%)	90 (70%)	0.2
Yes	54 (37%)	39 (30%)	
Secretagogue			
No	116 (80%)	103 (80%)	>0.9
Yes	29 (20%)	26 (20%)	

**Table 3:** Variables after the initiation of SGLT2 inhibitors ^1^Data are presented as n (%) or median (interquartile range) ^2^Pearson's chi-squared test A p-value of <0.05 was considered statistically significant BMI, body mass index; GFR, glomerular filtration rate; SGLT2, sodium-glucose co-transporter 2

Variable	Baseline	Last visit	p-Value^2^
HbA1c (%)	8.80% (7.60, 10.70)	8.30% (7.10, 9.40)	
GFR (mL/min/1.73 m^2^)	80 mL/min/1.73 m^2 ^(58, 95)	81 mL/min/1.73 m^2 ^(61, 97)	
BMI (kg/m^2^)	32.8 kg/m^2 ^(28.3, 36.8)	31.1 kg/m^2 ^(27.6, 34.7)	
Has the patient developed a urinary tract infection after SGLT-inhibitor use?	Female, N = 145^1^	Male, N = 129^1^	
No	116 (81%)	120 (93%)	0.004
Yes	27 (19%)	9 (7.0%)	
Responsible organism	N (%)		
Bacterial	30 (83.3)		
Fungal	6 (16.7)		

Men exhibited a lower risk of UTI development than women (HR, 0.38; 95% CI: 0.15, 0.96). Additionally, patients with a history of UTIs had a higher risk of experiencing recurrent infections after SGLT2 inhibitor initiation compared with those with no such history (HR: 3.32, 95% CI: 1.56, 7.09). Moreover, patients with a high GFR before SGLT2 inhibitor use had a lower risk of developing UTIs (HR, 0.98; 95% CI: 0.97, 0.99) (Table 4).

**Table 4:** Risk factors for urinary tract infection among patients with diabetes taking SLGT2 inhibitors Log-rank test was used. A p-value of <0.05 was considered statistically significant BMI, body mass; CI, confidence interval; GFR, glomerular filtration rate; HR, hazard ratio index

Characteristic	HR	95% CI	p-Value
Sex			
Female	—	—	
Male	0.38	0.15, 0.96	0.042
Prior history of urinary tract infection			
No	—	—	
Yes	3.32	1.56, 7.09	0.002
Baseline GFR	0.98	0.97, 0.99	0.004
BMI	0.98	0.92, 1.04	0.5

## Discussion

SGLT2 inhibitors have emerged as notable therapeutic options for the management of T2DM. However, a significant concern associated with its use is the elevated risk of genitourinary infections, which has been consistently reported in various studies [4]. This study aimed to evaluate the tolerability of SGLT2 inhibitors in patients with T2DM. Our findings showed that of the 275 patients (median age: 64 years) with T2DM, 36 (13.1%) who received SGLT2 inhibitors developed genitourinary infections. Additionally, the history of genital infections and UTIs varied significantly between men and women. Consistent with the results of multiple similar studies conducted globally and in Saudi Arabia [7-9], women had a higher frequency of history of both UTIs and genital infections than men.

**Figure 1:**
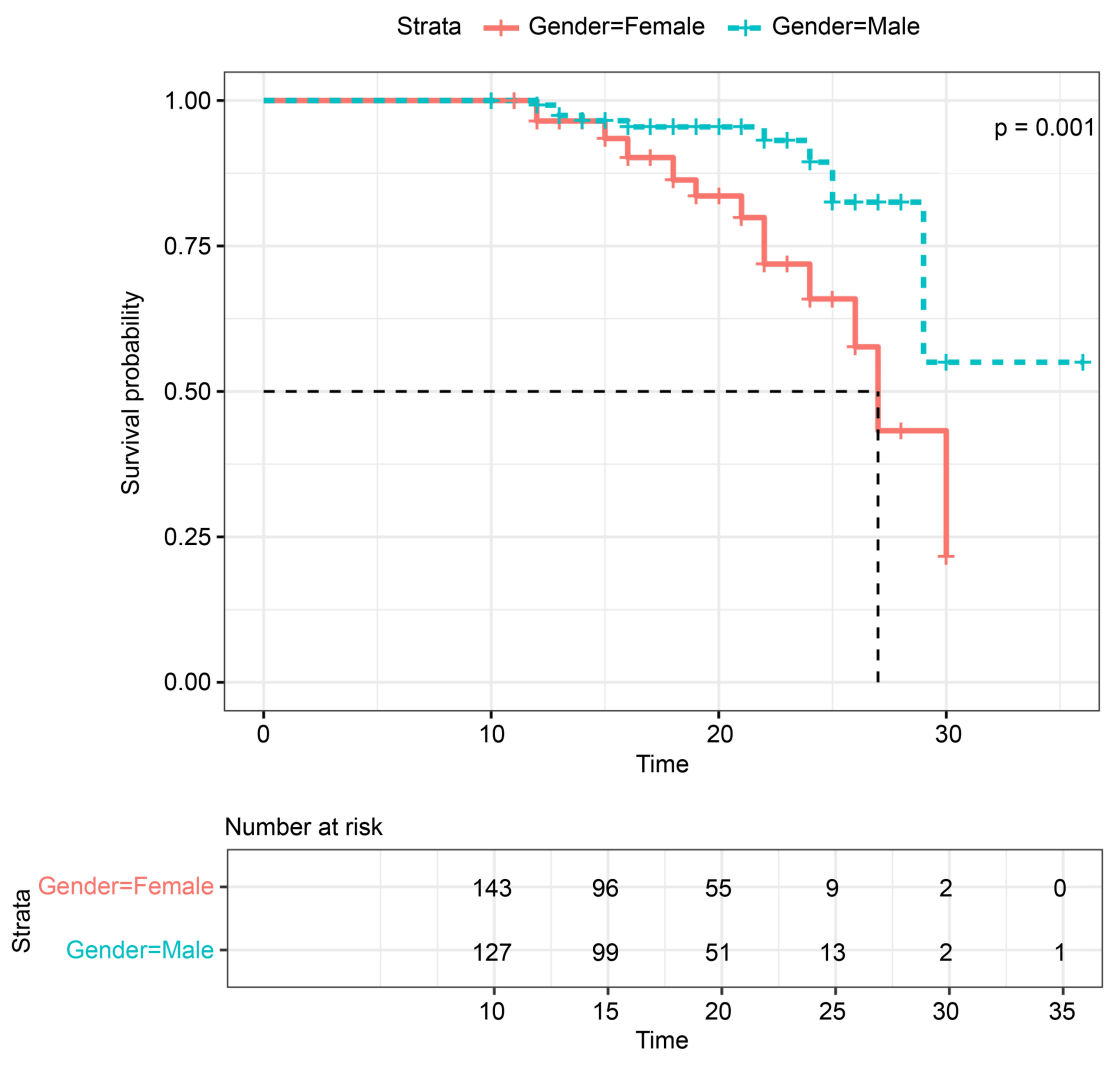
Kaplan–Meier curve for the development of urogenital infections associated with SGLT2 inhibitor The curve displays the outcomes of the log-rank test, indicating notable sex differences in the likelihood of developing urinary tract infections associated with SGLT2 inhibitor use. Women exhibited a significantly higher probability of urinary tract infection development (p<0.05). The x-axis represents time in months. A p-value of <0.05 was considered statistically significant. SGLT2, sodium-glucose co-transporter 2

These results indicate that sex may play a role in the susceptibility to these infections among patients with diabetes, as the anatomy and physiology of women may predispose them to the development of these infections, given the shorter urethra [10-12]. Furthermore, our study demonstrated that women were more likely to develop UTIs after SGLT2 inhibitor use than men. This finding suggests that the use of SGLT2 inhibitors may have a differential impact on the risk of UTI development according to sex, which may be explained by the aforementioned anatomical and physiological differences. SGLT2 inhibitors block the reabsorption of glucose in the kidneys and, therefore, promote the excretion of glucose in the urine. This provides a fertile environment for pathogens to replicate and infect the host [13-16]. The graphical representations of the data further support the observed sex differences regarding the risk of UTI development. Figure. 1 illustrates that women had a significantly higher probability of developing UTIs when using SGLT2 inhibitors than men, as indicated by the log-rank test. This finding underscores the need for sex-specific analyses and interventions to mitigate the elevated risk of UTIs in women.

In our study, patients with a history of UTIs had a higher risk of developing UTIs following SGLT2 inhibitor initiation than those with no history of UTIs. This result is consistent with the results of studies conducted globally [8,17-19] and indicates the need to consider UTIs as a major predictor of future infections. Additionally, patients with a higher GFR before SGLT2 inhibitor initiation had a lower risk of developing UTIs, suggesting that renal function may influence UTI susceptibility.

Although the current study reported a UTI development frequency of 13.1% in patients with diabetes treated with SGLT2 inhibitors, it is still lower than the reported national and global incidences, which can be partly explained by the smaller study population. In a study conducted in Manila in 2020, the incidence of genitourinary infections among 253 patients with T2DM who were taking SGLT2 inhibitors was 21.78% at the six-month follow-up [20]. Another study conducted in 2022 by Uitrakul et al. in Thailand demonstrated an increased incidence of UTIs in patients using SGLT2 inhibitors, with an overall rate of 33.49% [9]. Moreover, in Saudi Arabia, the rate of UTI development among patients with T2DM was 25.3% [8]. Additionally, a systematic review showed that the incidence of UTI development among patients with diabetes was 14.4%, which was higher in patients taking SGLT2 inhibitors (28.2%) [21].

Numerous studies have explored the association between SGLT2 inhibitors and the risk of UTIs. The conflicting findings among these studies highlight the complexity of assessing the relationship between SGLT2 inhibitors and the risk of UTIs. Factors such as the study design, patient population, and follow-up duration may have contributed to the observed discrepancies. A study by Li et al. in 2016 reported that the use of SGLT2 inhibitors was associated with an increased likelihood of UTI development compared with alternative treatments. Specifically, dapagliflozin uniquely demonstrated a dose-dependent relationship with the risk [22]. Conversely, Alkabbani et al. reported that SGLT2 inhibitor use led to no significant increase in the frequency of UTIs [23].

Multiple meta-analyses investigating SGLT2 inhibitors have reported inconsistent results regarding the risk of genitourinary infections. Many studies [4, 24-26] have documented elevated risks of UTIs and genital infections associated with the use of SGLT2 inhibitors. However, our study population demonstrated a lower risk of genitourinary infections than that previously reported. Based on our findings, we propose that glycosuria may not be the sole factor contributing to an increased risk of such infections, especially in men [27].

In a study by Butt et al. conducted in Saudi Arabia to examine the effects of empagliflozin [28], genitourinary tract infections occurred during the third follow-up in only six (2%) of the 305 participants. One of the theories proposed by Butt et al. to explain the lower incidence of UTIs was the differences in hygiene practices among different populations. Specifically, in the Middle East, washing with water after using the toilet is more common compared with Western countries, where the prevalent method is the use of tissue paper.

This study has certain limitations primarily due to its retrospective design. First, relying exclusively on hospital records to identify UTIs may lead to underreporting, as not all individuals with UTIs seek hospital care. Additionally, patients with long-standing T2DM and conditions such as neurogenic bladder may fail to recognize UTI symptoms and, consequently, might not present for medical evaluation or treatment. Second, the relatively short follow-up period may not adequately capture long-term effects or the sustainability of the observed outcomes. Third, the sample size of 275 participants, drawn from a single-center cohort, was relatively small, which limited the study's statistical power and the precision of its findings. These factors may impact the accuracy and generalizability of the results.

Despite these limitations, the study offers several strengths. It provides valuable real-world insights into the relationship between SGLT2 inhibitors and the risk of UTIs in patients with T2DM. The use of detailed hospital records allowed for a rigorous assessment of clinical outcomes, ensuring reliable documentation of diagnoses and treatment courses. Additionally, the focus on a specific patient cohort adds depth to the understanding of this issue, offering a foundation for future research. These findings can help guide clinical decision-making and highlight the importance of monitoring for UTIs in patients prescribed SGLT2 inhibitors.

Future research should consider prospective study designs and incorporate data from multiple healthcare centers to provide a more robust and comprehensive understanding of UTI risk associated with SGLT2 inhibitors across diverse patient populations.

## Conclusions

This study demonstrated no discernible increase in the risk of UTI development associated with the use of SGLT2 inhibitors in patients with T2DM, compared with the reported rates of UTIs, thus supporting the safety profile of SGLT2 inhibitors. The lower rates observed in our study and other local studies of SGLT inhibitor-associated UTIs compared with those in Western countries could be due to differences in hygiene practices. While this study contributes to the growing body of evidence, future multicenter studies are crucial to comprehensively assess the long-term effects and further strengthen our understanding of the relationship between SGLT2 inhibitors and UTI risk in patients with diabetes.
